# Urotensin-II System in Genetic Control of Blood Pressure and Renal Function

**DOI:** 10.1371/journal.pone.0083137

**Published:** 2013-12-31

**Authors:** Radoslaw Debiec, Paraskevi Christofidou, Matthew Denniff, Lisa D. Bloomer, Pawel Bogdanski, Lukasz Wojnar, Katarzyna Musialik, Fadi J. Charchar, John R. Thompson, Dawn Waterworth, Kijoung Song, Peter Vollenweider, Gerard Waeber, Ewa Zukowska-Szczechowska, Nilesh J. Samani, David Lambert, Maciej Tomaszewski

**Affiliations:** 1 Department of Cardiovascular Sciences, University of Leicester, Leicester, United Kingdom; 2 Department of Internal Medicine, Metabolic Disorders and Hypertension, Medical University of Poznan, Poznan, Poland; 3 Department of Urology and Oncological Urology, Medical University of Poznan, Poznan, Poland; 4 School of Science and Engineering, University of Ballarat, Ballarat, Australia; 5 GlaxoSmithKline, Philadelphia, Pennsylvania, United States of America; 6 Department of Medicine, Centre Hospitalier Universitaire Vaudois, Lausanne, Switzerland; 7 Department of Internal Medicine, Diabetology and Nephrology, Medical University of Silesia, Zabrze, Poland; University of Maryland School of Medicine, United States of America

## Abstract

Urotensin-II controls ion/water homeostasis in fish and vascular tone in rodents. We hypothesised that common genetic variants in urotensin-II pathway genes are associated with human blood pressure or renal function. We performed family-based analysis of association between blood pressure, glomerular filtration and genes of the urotensin-II pathway (urotensin-II, urotensin-II related peptide, urotensin-II receptor) saturated with 28 tagging single nucleotide polymorphisms in 2024 individuals from 520 families; followed by an independent replication in 420 families and 7545 unrelated subjects. The expression studies of the urotensin-II pathway were carried out in 97 human kidneys. Phylogenetic evolutionary analysis was conducted in 17 vertebrate species. One single nucleotide polymorphism (rs531485 in urotensin-II gene) was associated with adjusted estimated glomerular filtration rate in the discovery cohort (p = 0.0005). It showed no association with estimated glomerular filtration rate in the combined replication resource of 8724 subjects from 6 populations. Expression of urotensin-II and its receptor showed strong linear correlation (r = 0.86, p<0.0001). There was no difference in renal expression of urotensin-II system between hypertensive and normotensive subjects. Evolutionary analysis revealed accumulation of mutations in urotensin-II since the divergence of primates and weaker conservation of urotensin-II receptor in primates than in lower vertebrates. Our data suggest that urotensin-II system genes are unlikely to play a major role in genetic control of human blood pressure or renal function. The signatures of evolutionary forces acting on urotensin-II system indicate that it may have evolved towards loss of function since the divergence of primates.

## Introduction

Urotensin-II (U-II) is an eleven amino-acid peptide first extracted from neurophysis (neuro-secretory organ) of Goby fish (*Gillichthys mirabilis*) [1]. Native to the Gulf of California, Goby fish is known for its exceptional ability to withstand profound changes in temperature and osmolality of the aquatic environment [2]. One of the mechanisms involved in Goby's adaptation to rapidly changing osmolality is U-II [3]. In response to alternations in the salinity, fish modulate expression of U-II and its partner molecules in gills epithelium, urinary bladder and intestine [4]. Up-regulation of U-II pathway in Goby fish translates into inhibition of NaCl transport in a concentration dependent manner [4]. These data indicate that U-II system contributes to fish osmoregulation and ion-exchange; both of which are regulated by the mammalian kidney.

In mammals including mouse, rat, dog, pig, marmoset, cynomolgus monkey and human, U-II signaling pathway genes are expressed in the kidney [5]–[10]. In fact, kidneys have been hypothesised to act as a source of systemic production of U-II [11], [12]. Data from experimental models suggest that circulating levels of U-II may influence renal function in rodents. Indeed, in rats U-II associated not only with glomerular filtration rate (GFR) (through influence on renal blood flow) but also with renal natriuresis [13], [14].

U-II also is the most potent mammalian vasoconstrictor *in vitro* [5]. Elevated serum levels of U-II were linked to human hypertension, impaired renal function and diabetes but the trials of pharmacological inhibition of the system failed to provide evidence of its utility in therapy of elevated blood pressure (BP) [8], [15]–[20].

Systematic genetic association analysis may not only illuminate previously unexpected mechanisms underpinning biological processes but may also help to explain conflicting data from experimental models and *in vitro* physiological studies. However, data from systematic genetic analysis of U-II pathway are not available. Indeed, previous association studies were conducted on single functional polymorphic variants of this pathway and suffered from small sample size, insufficient genetic coverage and lack of appropriate replication [21]–[23].

Given the body of evidence (collected primarily from animal experiments) we hypothesised that genetic variation in U-II system on DNA and mRNA level may be associated with GFR and BP in humans. Using a large sample of both families and biologically unrelated individuals we conducted first systematic genetic analysis of U-II system in relation to human BP and estimated GFR (eGFR). This was followed by comparative expression analysis of U-II pathway genes in the hypertensive and normotensive human kidneys and evolutionary analysis of the system in vertebrates.

## Materials and Methods

### Genetic association study

#### Populations and phenotypes

The discovery cohort - Genetic Regulation of Arterial Pressure of Humans in the Community (GRAPHIC) Study consists of 2037 subjects from 520 white British pedigrees recruited from the general population in Leicestershire (UK) [24].

A total of 3521 individuals from five populations were used as a wet lab-based replication resource. We used two collections of pedigrees - Silesian Hypertension Study (SHS) with 629 individuals from 207 nuclear families recruited in Silesia (south of Poland) [25] and 213 families (703 subjects) from Silesian Cardiovascular Study (SCS) [26]. A total of 2189 biologically unrelated individuals collected in the replication resource were derived from Young Men Cardiovascular Association Study 1 (YMCA1) (n = 1157), Young Men Cardiovascular Association Study 2 (YMCA2) (n = 597) and SCS extension (n = 435) [26]. Additional *in silico* replication was conducted in 5356 individuals from the Swiss CoLaus Study [27].

All individuals were of white European ancestry.

Phenotyping of all individuals included taking clinical history (using anonymous coded questionnaires), physical examination, anthropometric measurements (weight, height) and biochemical tests (serum level of creatinine).

Estimated glomerular filtration rate was calculated from serum creatinine using Modification of Diet in Renal Disease (MDRD) formula: GFR (mL/min/1.73 m^2^) = 186×(Serum creatinine)^−1.154^×(Age)^−0.203^×(0.742 if female): in all individuals >18years old [28].

24-hour urinary excretion of sodium and potassium were available for individuals from the GRAPHIC Study.

In subjects from the GRAPHIC Study Ambulatory BP was measured (at 30-minute intervals between 08:00 and 21:59 [day time] and at 1-hour intervals between 22:00 and 07:59 [night time]) using a Spacelabs 90207 monitor (Spacelabs, Wokingham, UK) for approximately 26 hours. The first 2 hours of each recording were excluded from further analysis [29].

Clinic BP was recorded in triplicate in a sitting position using digital BP monitors (The GRAPHIC Study, SCS, CoLaus) or sphygmomanometers (SHS, YMCA1 and YMCA2) with an appropriate size cuff after necessary rest [30]. The results of all 3 measurements were averaged in SHS, SCS, YMCA1 and YMCA2; in the GRAPHIC Study and CoLaus only second and third measurements were averaged [27].

In GRAPHIC Study, SHS, and SCS the effect of antihypertensive treatment on BP was corrected for using a semi-parametric algorithm [31]. In brief, adjustment value is calculated by subtracting the mean of the regression based residuals of treated individuals from the mean residuals of non-treated subjects (matched for BP readings). In individuals on treatment, treatment adjusted BP value is then a sum of the measured BP and adjustment value.

In CoLaus, YMCA1 and YMCA2 for individuals taking antihypertensive therapies, blood pressure was imputed by adding 15 mm Hg and 10 mm Hg for systolic BP (SBP) and diastolic BP (DBP), respectively [31].

#### Single nucleotide polymorphisms and genes of U-II pathway

**Figure 1:**
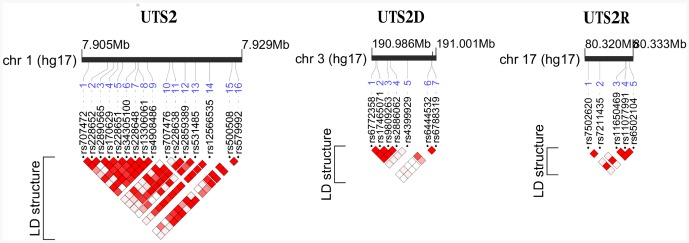
Urotensin-II pathway genes and hypertension. Figure present structure of UTS2, UTS2D and UTS2R genes - linkage disequilibrium (LD) map based on r^2^ coefficients [intensity of colour: r^2^ = 1 – maximal LD (dark red), r^2^ = 0 – no LD (white)] in the GRPAHIC Study.

Three genes of the pathway – urotensin-II gene (UTS2), urotensin-II related peptide gene (UTS2D) and urotensin-II receptor gene (UTS2R) were selected for genetic association analysis. A total of 28 tagging single nucleotide polymorphisms (SNP) in these loci (16 in UTS2, 7 in UTS2D and 5 in UTS2R) were chosen for genotyping in the GRAPHIC Study to capture the common genetic variation under the following criteria: minor allele frequency (MAF)>0.05 and r^2^>0.8 (Figure 1). The detailed description of SNPs of the pathway is in the Table S1 in file S1. Genotypes of these SNPs in the discovery cohort (the GRAPHIC) were partly derived from Illumina HumanCVD BeadChip (50K IBC array) [32] or obtained through direct TaqMan-based DNA analysis (on ABI PRISM 7900HT Sequence Detection System, Applied Biosystems). The SNP taken forward for replication was genotyped directly by TaqMan in SHS, SCS, YMCA1 and YMCA2 or extracted *in silico* from the Affymetrix Genome-Wide Human SNP Array used before in genome-wide analysis in CoLaus (IMPUTE version 0.5, imputation coefficient of accuracy 0.94) [29], [33]. The details of SNP selection and quality control criteria are included in Tables S1 and S2 in file S1.

#### Statistical analysis

Heritability analysis of eGFR, clinic SBP and DBP in 2 cohorts of families (GRAPHIC Study, SCS families,) was performed using variance component analysis algorithm implemented in SOLAR (Sequential Oligogenic Linkage Analysis Routines) v2.0 software after adjustment for age, age^2^ and sex [34].

The primary analysis of genetic associations in families was conducted using Family Based Association Test (FBAT). Associations between SNPs of the candidate pathway and age, age^2^, sex and antihypertensive treatment (where appropriate) adjusted clinic SBP, DBP, eGFR, 24 hour urinary sodium excretion, 24-hour urinary potassium excretion, mean 24-hour SBP, mean 24-hour DBP were examined under additive model of inheritance under the null hypothesis of no linkage and no association. This method of analysis is immune to population stratification [35]. Generalized Estimating Equations (GEE) were then used to obtain quantitative measures (β-coefficients and standard errors) of the detected associations to facilitate the meta-analysis of the studies [36].

The analysis of association between U-II pathway SNP(s) in cohorts of biologically unrelated subjects was performed using PLINK-based [37] multiple linear regression model under additive model of inheritance after adjustment for age sex and BP-lowering effect of antihypertensive therapy (where appropriate).

In the discovery cohort the correction for multiple testing (28 SNPs and 7 phenotypes) was calculated using a spectral decomposition of linkage disequilibrium statistics as proposed by Nyholt [38]. Specifically, based on a calculated number of independent genetic markers and phenotypes a threshold of p = 0.0007 was calculated to keep type 1 error rate below 10%. The details of this calculation are included in file S1.

Meta-analysis in all replication cohorts was conducted using a fixed effect, inverse variance model implemented in Metan script (STATA) [39]. Heterogeneity between studies was examined using χ^2^test.

### Gene expression analysis

U-II pathway gene expression was examined in two independent collections of human kidneys: Silesian Renal Tissue Bank (SRTB, n = 62, Poland) and the Western Poland Kidney Project (WPKP, n = 35, Poland) [40], [41]. Both collections come from well phenotyped patients undergoing unilateral nephrectomy for non-invasive renal cancer. A sample of human kidney tissue was collected from unaffected by cancer (apparently healthy) pole of the kidney and preserved in RNAlater (Ambion) and −70C prior to gene expression studies. Each individual underwent phenotyping similar to the protocol used in SHS [40]. Hypertension was defined as BP values ≥140/90 mmHg measured on at least two separate occasions and/or taking anti-hypertensive medication.

Renal mRNA expression was quantified using designed TaqMan gene expression assays (Applied Biosystems) and normalized to the expression of a housekeeping control gene (β-microglobulin). All experiments were performed in duplicates. Multiple regression models (with gene expression as a dependent variable and age, sex, cohort and hypertension status) were used to examine adjusted difference in gene expression between normotensive and hypertensive subjects.

### Evolutionary analysis

The analysis of evolutionary pressure acting on coding sequences of U-II pathway genes was conducted using ratios of non-synonymous to synonymous codon substitutions [42]. Synonymous substitutions do not lead to changes in the amino acid composition of a protein and are generally evolutionary neutral. Non-synonymous substitutions lead to changes in the amino acid composition of a protein and therefore provide an evolutionary disadvantage or advantage for the carrier. DNA and peptide sequences of UTS2, UTS2D and UST2R for all available vertebrate species were obtained from ENSEMBL database http://www.ensembl.org/index.html. Where more than one splice variant was available, the longest was used as the reference sequence. Multiple peptide sequences were aligned using Teecoffee@igs web server (http://igs-server.cnrs-mrs.fr/Tcoffee/tcoffee_cgi/i ndex.cgi) and manually inspected for accuracy [43]. Sequences missing the biologically important C-terminal fragments of U-II or urotensin-II related peptide (URP) or sequences substantially shorter than the average (introducing large gaps into the alignment) were removed from further analysis. The ultimate peptide alignments were used to guide complementary DNA (cDNA) alignment using RevTrans 1.4 web server (http://www.cbs.dtu.dk/services/RevTrans/) [44]. The cDNA alignments were then used in evolutionary analysis conducted on Adaptive Evolution Server (http://datamonkey.org). HKY85 nucleotide substitution model was chosen using a genetic algorithm available on http://datamonkey.org. Other parameters of the model (substitution rates, codon frequencies and distances) were estimated using maximum likelihood procedures [45]. Phylogenetic line specific evolutionary models were fitted using GABranch algorithm available on http://datamonkey.org server.

### Ethical approval

All subjects gave informed, written consent for participation and the studies were approved by the appropriate Bioethical Committees. These were: Leicestershire Research Ethics Committee - The GRAPHIC Study; Bioethical Committee of the Medical University of Silesia - SHS, SCS, YMCA1, YMCA2, SRTB; Institutional Ethics Committee of the University of Lausanne - CoLaus; Bioethical Committee of the Karol Marcinkowski's Medical University in Poznan - WPKP.

## Results

### Demographic characteristics

The basic clinical characteristics of populations used in the genetic association and gene expression analyses are presented in Table 1.

**Table 1:** Clinical characteristics of populations.

	GRAPHIC (2024)	SHS (629)	SCS families (703)	SCS extension panel (435)	YMCA1 (1157)	YMCA2 (597)	CoLaus (5356)	SRTB (62)	WPKP (35)
**Age (years)**	39.3±14.5	45.8±15.7	43.4±15.9	55.1±12.2	19.1±3.6	18.9±3.5	53.4±10.7	58.6±11.5	63.5±10.1
**BMI (kg/m^2^)**	26.1±4.6	26.8±4.6	26.2±4.8	27.1±3.9	22.8±3.0	22.6±3.0	25.8±4.6	27.3±4.0	26.1±5.1
**eGFR (mL/min/1.73 m^2^)**	83.9±14.8	68.9±14.1	82.8±19.4	75.5±17.6	131.0±24.0	124.5±19.1	78.4±15.6	-	-
**Clinic SBP (mmHg)**	127.1±17.9	138.4±19.9	136.5±20.7	125.5±19.4	117.9±13.2	119.0±13.5	128.5±17.9	-	-
**Clinic DBP (mmHg)**	79.1±11.0	88.0±11.9	78.2±13.0	73.0±11.0	74.2±7.9	74.4±8.2	79.4±10.8	-	-
**Hypertension (%, N)**	29 (577)	64 (401)	51 (361)	73 (316)	10 (120)	6 (40)	26 (1371)	61 (38)	74 (26)
**Antihypertensive treatment (%, N)**	7 (135)	43 (270)	36 (251)	62 (270)	2 (18)	1 (5)	18 (982)	61 (38)	74 (26)

Data are arithmetic means ± standard deviations or percentages and counts, where appropriate, GRAPHIC – Genetic Regulation of Arterial Pressure of Humans in the Community, SHS – Silesian Hypertension Study, SCS – Silesian Cardiovascular Study, YMCA – Young Men Cardiovascular Association study, SRTB – Silesian Renal Tissue Bank, WPKP – Western Poland Kidney Project, BMI – body mass index, eGFR – estimated glomerular filtration rate, SBP – systolic blood pressure, DBP – diastolic blood pressure.

### Heritability analysis

Taking advantage of the availability of data from family cohorts we estimated narrow-sense heritability for eGFR and BP in 733 families of white European ancestry. The age and sex adjusted eGFR showed very significant heritable component ranging from 38.4% in the GRAPHIC Study (p = 5.5×10^−25^) to 46.5% in the SCS families (p = 1.3×10^−11^) (Table 2). Both clinic SBP and clinic DBP showed also substantial heritable component estimated in the range of 30.5% (SBP in the GRAPHIC Study) to 47.1% (SBP in SCS families) (Table 2).

**Table 2:** Blood pressure and estimated glomerular filtration rate – heritability analysis.

Cohort	Heritability of eGFR		Heritability of clinic SBP		Heritability of clinic DBP	
	h^2^ (SE)	P-value	h^2^ (SE)	P-value	h^2^ (SE)	P-value
**GRAPHIC Study**	0.384 (0.038)	5.5×10^−25^	0.305 (0.043)	2.5×10^−13^	0.317 (0.041)	2.2×10^−15^
**SCS families**	0.466 (0.069)	1.3×10^−11^	0.471 (0.074)	3.5×10^−10^	0.371 (0.080)	1.7×10^−6^

h^2^ – narrow sense heritability, SE – standard error, P-value – level of statistical significance, GRAPHIC – Genetic Regulation of Arterial Pressure of Humans in the Community, SCS – Silesian Cardiovascular Study, eGFR – estimated glomerular filtration rate, SBP – systolic blood pressure, DBP - diastolic blood pressure.

### Genetic association analysis

The GRAPHIC based analysis of 28 tagging SNPs (in UTS2, UTS2D and UTS2R) and 7 phenotypes (eGFR, 24-hour urinary sodium excretion, 24-hour urinary potassium excretion, mean 24-hour SBP, mean 24-hour DBP, clinic SBP and clinic DBP) showed that only one SNP (rs531485) was significantly associated with eGFR after correction for multiple testing (p = 0.0005) (Tables S3–S9 in file S1). This SNP was taken forward for replication and genotyped directly in 2 family-based studies but showed no association with eGFR in either (SCS families - p = 0.6779, SHS - p = 0.6984) (Table 3). In cohorts of unrelated subjects rs531485 showed association with eGFR only in YMCA2 (p = 0.0130). However, the direction of this association was opposite to the one observed in the GRAPHIC Study. The rs531485 was not associated with adjusted eGFR in the CoLaus study (p = 0.9019). The meta-analysis of 8724 subjects from 6 populations showed no association between rs531485 and eGFR (p = 0.5834) (Table 3).

**Table 3:** Association between eGFR and minor allele of rs531485 in urotensin-II gene – fixed effect inverse variance meta-analysis in the replication resource.

Study	Informative subjects	β±SE	P-value	P-value*
**SCS families**	684	1.34±1.11	0.2288	
**SHS**	596	−0.54±0.73	0.4624	
**SCS replication panel**	430	−0.42±0.95	0.6564	
**YMCA1**	1119	−0.06±1.21	0.9612	
**YMCA2**	539	−3.29±1.32	0.0130	
**CoLaus Study**	5356	0.04±0.35	0.9019	
**Meta-analysis**	**8724**	**−0.15±0.28**	**0.5834**	**0.1494**

SCS – Silesian Cardiovascular Study, SHS – Silesian Hypertension Study, YMCA – Young Men Cardiovascular Association Study, informative subjects – number of subjects with available genotypic and phenotypic data, β – estimated effect size, SE – standard error of the estimate, P-value – level of statistical significance, P-value* - level of the statistical significance in the χ^2^ test of heterogeneity.

### Gene expression analysis

**Figure 2:**
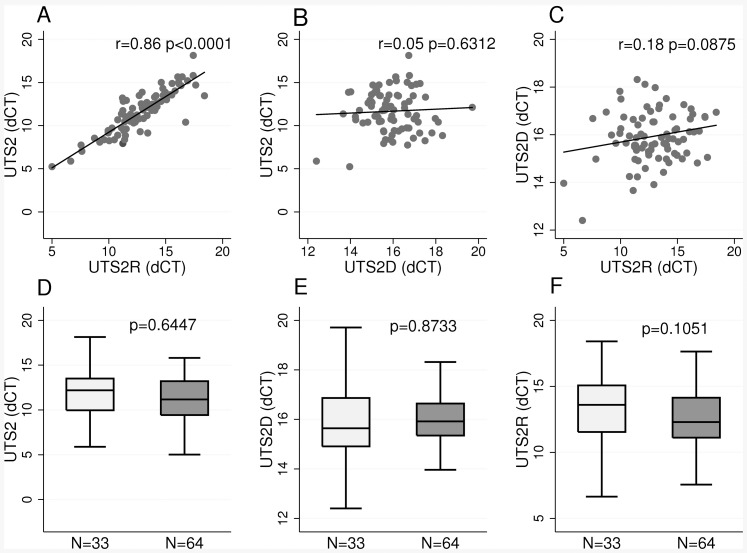
Renal expression of Urotensin-II pathway genes. Upper panel - linear correlations in renal expression (dCT) of UTS2, UTS2D, and UTS2R (r – correlation coefficient, p – level of statistical significance). Lower panel - comparison of UTS2, UTS2D and UTSR expression between normotensive (bright grey) and hypertensive (dark grey) kidneys (p-values from multiple regression adjusted for age, sex and cohort of origin).

The expression of UTS2 and its receptor UTS2R showed strong linear correlation in the human kidneys (r = 0.86, P<0.0001) (Figure 2). Neither UTS2 and UTS2D nor UTS2D and UTS2R showed significant linear correlation (r = 0.05, P = 0.6312), (r = 0.18, P = 0.0875), respectively (Figure 2).

The comparative expression analysis of U-II system genes between kidneys from normotensive and hypertensive subjects (altogether 97 subjects) revealed no statistically significant differences (UTS2: p = 0.6447; UTS2D: p = 0.8733; UTS2R: p = 0.1051) (Figure 2).

None of the 19 tagging SNPs genotyped in Silesian Renal Tissue Bank (SRTB) showed association with expression of renal mRNA of the relevant gene (Table S10 in file S1).

### Evolutionary analysis

Line specific evolutionary analysis of pre-pro urotensin-II sequences showed substantial differences across species with non-synonymous (dN) to synonymous substitution (dS) ratios ranging from 0.27 (strong conservation) to 1.6 (accumulation of mutations - lack of conservation). There was a clear division between phylogenetically older species and primates; they were separated by long period of diversifying selection (Figure S1 in file S1). Evolutionary analysis showed variable levels of purifying selection acting on UTS2D and UTS2R with no evidence of periods of diversifying selection (Figures S2 and S3 in file S1). Primates exhibit relatively weakest purifying selection on UTS2R across phylogeny.

## Discussion

Our data show that common genetic variation in U-II pathway is unlikely to play a major role in genetic regulation of human BP or eGFR. There is a significant co-regulation between U-II and its receptor in the human kidney but their expression is not associated with hypertension. Additionally, our data suggests that biological functions of U-II system may differ across species. This is supported by our evolutionary analysis showing diversifying selection (accumulating mutations) acting on the coding sequence of U-II since the divergence of primates.

Our association study is the first systematic analysis of the role of common variants in U-II system in regulation of human BP and eGFR. The results of this analysis are in essence negative – the initial signal of association between U-II and eGFR was not confirmed in the replication study of 8724 subjects. Previous studies on association between U-II system genes and cardiovascular phenotypes were performed on very small population samples and a majority of them lacked independent replication [21]–[23]. Our analysis based on a total of >10000 subjects is the largest association experiment on genetic variants within U-II pathway genes to date.

The results of our gene expression experiments in SRTB and Western Poland Kidney Project (WPKP) showed no evidence for association of the pathway with human hypertension. To the best of our knowledge this is the first gene expression analysis of the U-II system of this scale (almost 100 human kidneys) and the first direct comparative analysis of UTS2, UTS2D and UTS2R expression in human tissue from normotensive and hypertensive subjects. We confirmed that all three molecules of U-II pathway were expressed in human kidney and that UTS2 and its receptor (UTS2R) show strong correlation in expression. This suggests that renal transcripts of UTS2 and UTS2R are under tight functional co-regulation. A study by Matsushita et al. previously reported co-expression of UTS2 and UTS2R in 5 samples taken from human renal tissue [46]. This small sample size did not permit to confirm/exclude the presence of co-regulation between both transcripts [46]. No association between expression of U-II system in hypertension in our study is in odds to findings from Cheung et al. who showed raised plasma U-II levels in hypertensive patients [16]. However, the value of measuring serum levels of U-II by currently available radioimmunoassay has been questioned [47].

Strong evolutionary conservation of the C-terminal cyclic domain across different species was previously interpreted as an evidence for the importance of U-II in physiology [48]. Our evolutionary analysis showed very different signatures of evolution acting on coding sequences of UTS2, UTS2D and UTS2R across different species - dN/dS ratios ranged from 0.14 (purifying selection) to 1.15 (accumulation of mutations). The evolutionary pressures were weaker in UTS2 than UTS2D or UTS2R. Stronger conservation of UTS2R than UTS2 is consistent with its fundamental function in signaling of the pathway. UTS2 underwent a period of strong diversifying selection (accumulation of amino-acid changes) following divergence of primates. Similar trends are apparent in the analysis of UTS2R - purifying selection in primates was weaker than in the phylogenetically older vertebrates. Given the evolutionary difference, direct extrapolation of results obtained from lower vertebrates to primates should be made with caution. This could be supported by the presence of differences between functioning of the system in different species; e.g. rodent and primate UT act differently upon stimulation with an artificial ligand; native and non-native U-II exerts different effect on tissues [15], [49], [50].

Our data suggest that genetic variation within U-II system is not associated with BP or eGFR in the population of white European ancestry. The U-II system may play an important role in phylogenetically older animals but it appears to have slowly evolved towards loss of function since the divergence of primates. Our data indicate that U-II pathway is an unlikely target for further pharmacogenetic investigations.

## Supporting Information

File S1 **Tables S1–S10 and Figures S1–S3.** Table S1. SNPs in the U-II system genes - GRAPHIC Study. SNP – single nucleotide polymorphism, UTS2 – urotensin-II gene, UTS2D – urotensin-II related peptide gene, UTS2R – urotensin-II receptor gene, Chr – chromosome, position – genetic position on chromosome according to NCBI (CEU), MAF – minor allele frequency, MA – minor allele, 3′ – 3′ untranslated region, 5′ – 5′ untranslated region, TaqMan – genotyping using TaqMan probes (Applied Biosystems), 50K IBC – genotyped using 50k Illumina HumanCVD BeadChip. Table S2. Quality control of genotyping in the GRAPHIC Study. SNP – single nucleotide polymorphism, UTS2 – urotensin-II gene, UTS2D – urotensin-II related peptide gene, UTS2R – urotensin-II receptor gene, Chr – chromosome, position – genetic position on chromosome according to NCBI (CEU), MAF – frequency of the minor allele (calculated in parental generation for SCS families and SHS), HWE P-value – the level of statistical significance in Hardy-Weinberg equilibrium test. Table S3. Association between U-II system genes and estimated glomerular filtration rate in the GRAPHIC Study. SNP – single nucleotide polymorphism, “rs” – SNP identifier in the NCBI, UTS2 – urotensin-II gene, UTS2D – urotensin-II related peptide gene, UTS2R – urotensin-II receptor gene, MA – minor allele, MAF – frequency of the minor allele in the parental generation, Z – Z score – statistic calculated by the family-based association test showing the direction of association, P-value – level of statistical significance. Table S4. Association between U-II system genes and log10 transformed 24-hour urinary sodium excretion in the GRAPHIC Study. SNP – single nucleotide polymorphism, “rs” – SNP identifier in the NCBI, UTS2 – urotensin-II gene, UTS2D – urotensin-II related peptide gene, UTS2R – urotensin-II receptor gene, MA – minor allele, MAF – frequency of the minor allele in the parental generation, Z – Z score – statistic calculated by the family-based association test showing the direction of association, P-value – level of statistical significance. Table S5. Association between U-II system genes and log10 transformed 24-hour urinary potassium excretion in the GRAPHIC Study. SNP – single nucleotide polymorphism, “rs” – SNP identifier in the NCBI, UTS2 – urotensin-II gene, UTS2D – urotensin-II related peptide gene, UTS2R – urotensin-II receptor gene, MA – minor allele, MAF – frequency of the minor allele in the parental generation, Z – Z score – statistic calculated by the family-based association test showing the direction of association, P-value – level of statistical significance. Table S6. Association between urotensin-II system genes and mean 24-hour systolic blood pressure in the GRAPHIC Study. SNP – single nucleotide polymorphism, “rs” – SNP identifier in the NCBI, UTS2 – urotensin-II gene, UTS2D – urotensin-II related peptide gene, UTS2R – urotensin-II receptor gene, MA – minor allele, MAF – frequency of the minor allele in the parental generation, Z – Z score – statistic calculated by the family-based association test showing the direction of association, P-value – level of statistical significance. Table S7. Association between U-II system genes and mean 24-hour diastolic blood pressure in the GRAPHIC Study. SNP – single nucleotide polymorphism, “rs” – SNP identifier in the NCBI, UTS2 – urotensin-II gene, UTS2D – urotensin-II related peptide gene, UTS2R – urotensin-II receptor gene, MA – minor allele, MAF – frequency of the minor allele in the parental generation, Z – Z score – statistic calculated by the family-based association test showing the direction of association, P-value – level of statistical significance. Table S8. Association between U-II system genes and clinic systolic blood pressure in the GRAPHIC Study. SNP – single nucleotide polymorphism, “rs” – SNP identifier in the NCBI, UTS2 – urotensin-II gene, UTS2D – urotensin-II related peptide gene, UTS2R – urotensin-II receptor gene, MA – minor allele, MAF – frequency of the minor allele in the parental generation, Z – Z score – statistic calculated by the family-based association test showing the direction of association, P-value – level of statistical significance. Table S9. Association between urotensin-II system genes and clinic diastolic blood pressure in the GRAPHIC Study. SNP – single nucleotide polymorphism, “rs” – SNP identifier in the NCBI, UTS2 – urotensin-II gene, UTS2D – urotensin-II related peptide gene, UTS2R – urotensin-II receptor gene, MA – minor allele, MAF – frequency of the minor allele in the parental generation, Z – Z score – statistic calculated by the family-based association test showing the direction of association, P-value – level of statistical significance. Table S10. Association between SNPs in U-II pathway genes and renal expression of UTS2, UTS2D and UTS2R in kidneys from SRTB. SNP – single nucleotide polymorphism, “rs” – SNP identifier in the NCBI, UTS2 – urotensin-II gene, UTS2D – urotensin-II related peptide gene, UTS2R – urotensin-II receptor gene, MA – minor allele, MAF – frequency of the minor allele, β – effect size per each copy of minor allele, SE – standard error, P-value – level of statistical significance (adjusted for age and sex). Figure S1. Molecular evolution of urotensin-II. Green lines represent periods of diversifying selection with non-synonymous to synonymous substitution ratio of 1.6 (accumulation of non-synonymous mutations leading to changes in the protein sequence), orange lines - moderate purifying selection with non-synonymous to synonymous substitution ratio of 0.6; red lines – periods of strong purifying selection with non-synonymous to synonymous substitution ratio of 0.27. Figure S2. Molecular evolution of urotensin-II related peptide. Green lines represent periods of weak purifying selection with non-synonymous to synonymous substitution ratio of 0.6, orange lines represent strong purifying selection with non-synonymous to synonymous substitution ratio of 0.2; red lines represent periods of very strong purifying selection with non-synonymous to synonymous substitution ratio of 0.03. Figure S3. Molecular evolution of urotensin-II receptor. Green lines represent periods of moderately strong purifying selection with non-synonymous to synonymous substitution ratio of 0.25, orange lines represent moderate purifying selection with non-synonymous to synonymous substitution ratio of 0.18; red lines represent periods of very strong purifying selection with non-synonymous to synonymous substitution ratio of 0.04.
(DOCX)
