# Supplementary material for: Urotensin-II System in Genetic Control of Blood Pressure and Renal Function
Source: PLoS One. 2013 Dec 31;8(12):e83137. doi: 10.1371/journal.pone.0083137 (PMC3877024; doi:10.1371/journal.pone.0083137)
Supplement: File S1 — Tables S1–S10 and Figures S1–S3. Table S1. SNPs in the U-II system genes - GRAPHIC Study. SNP – single nucleotide polymorphism, UTS2 – urotensin-II gene, UTS2D – urotensin-II related peptide gene, UTS2R – urotensin-II receptor gene, Chr – chromosome, position – genetic position on chromosome according to NCBI (CEU), MAF – minor allele frequency, MA – minor allele, 3′ – 3′ untranslated region, 5′ – 5′ untranslated region, TaqMan – genotyping using TaqMan probes (Applied Biosystems), 50K IBC – genotyped using 50k Illumina HumanCVD BeadChip. Table S2. Quality control of genotyping in the GRAPHIC Study. SNP – single nucleotide polymorphism, UTS2 – urotensin-II gene, UTS2D – urotensin-II related peptide gene, UTS2R – urotensin-II receptor gene, Chr – chromosome, position – genetic position on chromosome according to NCBI (CEU), MAF – frequency of the minor allele (calculated in parental generation for SCS families and SHS), HWE P-value – the level of statistical significance in Hardy-Weinberg equilibrium test. Table S3. Association between U-II system genes and estimated glomerular filtration rate in the GRAPHIC Study. SNP – single nucleotide polymorphism, “rs” – SNP identifier in the NCBI, UTS2 – urotensin-II gene, UTS2D – urotensin-II related peptide gene, UTS2R – urotensin-II receptor gene, MA – minor allele, MAF – frequency of the minor allele in the parental generation, Z – Z score – statistic calculated by the family-based association test showing the direction of association, P-value – level of statistical significance. Table S4. Association between U-II system genes and log10 transformed 24-hour urinary sodium excretion in the GRAPHIC Study. SNP – single nucleotide polymorphism, “rs” – SNP identifier in the NCBI, UTS2 – urotensin-II gene, UTS2D – urotensin-II related peptide gene, UTS2R – urotensin-II receptor gene, MA – minor allele, MAF – frequency of the minor allele in the parental generation, Z – Z score – statistic calculated by the family-based association [file pone.0083137.s001.docx]

**Urotensin-II system in genetic control of blood pressure and renal function**

**Supplementary material**

Radoslaw Debiec^a^, Paraskevi Christofidou^a^, Matthew Denniff^a^, Lisa D. Bloomer^a^, Pawel Bogdanski^b^, Lukasz Wojnar^c^, Katarzyna Musialik^b^, Fadi J. Charchar^d^, John R. Thompson^a^, Dawn Waterworth^e^, KijoungSong^e^, Peter Vollenweider^f^, Gerard Waeber^f^, Ewa Zukowska-Szczechowska^g^, Nilesh J. Samani^a^, David Lambert^a^ and Maciej Tomaszewski^a^

1. Department of Cardiovascular Sciences, University of Leicester, UK;
2. Department of Internal Medicine, Metabolic Disorders and Hypertension, Medical University of Poznan, Poland;
3. Department of Urology and Oncological Urology, Medical University of Poznan, Poland;
4. School of Science and Engineering, University of Ballarat, Australia;
5. GlaxoSmithKline, Philadelphia, USA;
6. Department of Medicine, Centre Hospitalier Universitaire Vaudois, Lausanne, Switzerland;
7. Department of Internal Medicine, Diabetology and Nephrology, Medical University of Silesia, Zabrze, Poland

**Correspondence:**

Maciej Tomaszewski, MD, FAHA, FRCP

Department of Cardiovascular Sciences, University of Leicester, Glenfield Hospital,

Leicester LE3 9QP, UK

Phone: +44 116 204 4752;

Fax: 44-116-287-5792;

E-mail: mt142@le.ac.uk

**Methods**

**Genetic variants within U-II pathway genes genotyped in the study**

A total of 28 SNPs in the U-II pathway were selected for genotyping in the GRAPHIC Study.

Altogether, 16 SNPs were genotyped in the GRAPHIC Study in UTS2. Of these 11 SNPs were genotyped using 50k Illumina HumanCVD BeadChip[[1](#_ENREF_1)] and another 5 SNPs were added based on HapMap derived tagging algorithm. The following criteria were used: MAF>5% and r^2^>0.8. The additional 5 SNPs were genotyped using TaqMan® SNP Genotyping Assay (Applied Biosystems) (Table S1).

Only 1 SNP from UTS2D was genotyped using the 50k Illumina HumanCVD BeadChip array. Therefore, 6 additional SNPs were selected from HapMap (CEU) using tagging algorithm under the following criteria (MAF>5% and r^2^>0.8). The additional 6 SNPs were genotyped using TaqMan® SNP Genotyping Assay (Applied Biosystems) (Table S1).

Of the 5 UTS2R SNPs genotyped, 2 (rs11077991, rs6502104) were chosen from the HapMap (CEU) using a tagging algorithm under the following criteria: MAF>5% and r^2^>0.8. The additional SNPs in this gene (rs7502620, rs7211435, rs11650469) were chosen based on their putative biological function (as assessed by Brainarray SNP Function Annotation Portal (<http://brainarray.mbni.med.umich.edu/Brainarray/Database/SearchSNP/snpfunc.asp>). All SNPs in UTS2R were genotyped using TaqMan® SNP Genotyping Assay (Applied Biosystems) (Table S1).

**Quality control of genotyping**

Prior to statistical analysis, genotyping quality of all SNPs was thoroughly checked. Based on visual inspection of scatter plots SNPs with poor quality genotyping were re-genotyped. The familial structure of the GRAPHIC Study, SHS and SCS allowed for Mendelian inconsistencies to be identified. All SNPs genotyped in UTS2, UTS2D and UTS2R had MAF similar to those reported in National Centre for Biotechnology Information (NCBI) database for Caucasian population. All SNPs were common (MAF between 10.8% and 45.9%) except rs2890565 (MAF – 3.2%). The quality of genotyping was generally good and the proportion of missing genotypes was small. No SNPs violated the threshold of Hardy Weinberg equilibrium (p<0.001). The details of quality control results for each SNP are presented in Table S2.

**Correction for multiple testing**

To calculate a correction for multiple testing in the discovery cohort a spectral decomposition of linkage disequilibrium statistics, proposed by Nyholt was used.[[2](#_ENREF_2)] Genetic markers with close physical distances show high levels of correlation arising from their origin on a common ancestral haplotype.[[3](#_ENREF_3)] The same is true for closely correlated phenotypes. Thus, use of Bonferroni correction, assuming independence of all tested SNPs and phenotypes, would be overly conservative.[[4](#_ENREF_4)] The spectral decomposition identifies the number of truly independent statistical tests in the dataset after taking into account correlation between the tested variables.[[2](#_ENREF_2)] Nyholt’s correction calculates eigenvalues from a pair-wise correlation matrix of examined variables. The variance of the eigenvalues represents the overall correlation in the dataset (ranging from 0 [when all variables are independent] to n [n - total number of variables]). A ratio of observed to maximal eigenvalues variance represents a proportional reduction in the number of variables. This allows for the identification of the true number of independent variables in the data. The statistical significance threshold is then generated based on the number of independent statistical tests.

Spectral decomposition of SNPs genotyped in the discovery cohort revealed; 13.89, 5.84 and 3.39 fully independent genetic markers in UTS2, UTS2D and UTS2R, respectively. The corresponding level of statistical significance in the primary association analysis was calculated at p=0.0007 (α<0.1).

**Table S1.**

| **SNP** | **Gene** | **Chr** | **Position (bp)** | **MAF** | **Location** | **Genotyping format** |
| --- | --- | --- | --- | --- | --- | --- |
| **rs707472** | UTS2 | 1 | 7906008 | 0.167 | 3' | 50K IBC |
| **rs228652** | UTS2 | 1 | 7908888 | 0.264 | Intron | 50K IBC |
| **rs2890565** | UTS2 | 1 | 7909737 | 0.295 | Exon | 50K IBC |
| **rs170629** | UTS2 | 1 | 7910391 | 0.306 | Intron | TaqMan |
| **rs228651** | UTS2 | 1 | 7911099 | 0.278 | Intron | TaqMan |
| **rs34305100** | UTS2 | 1 | 7913029 | 0.181 | Exon | TaqMan |
| **rs228648** | UTS2 | 1 | 7913430 | 0.403 | Exon | TaqMan |
| **rs13306061** | UTS2 | 1 | 7913445 | 0.167 | Exon | TaqMan |
| **rs4908486** | UTS2 | 1 | 7914835 | 0.3 | 5' | 50K IBC |
| **rs707476** | UTS2 | 1 | 7918106 | 0.264 | 5' | 50K IBC |
| **rs228638** | UTS2 | 1 | 7918598 | 0.486 | 5' | 50K IBC |
| **rs2859389** | UTS2 | 1 | 7920423 | 0.278 | 5’ | 50K IBC |
| **rs531485** | UTS2 | 1 | 7921952 | 0.181 | 5’ | 50K IBC |
| **rs12566535** | UTS2 | 1 | 7924094 | 0.222 | 5' | 50K IBC |
| **rs500508** | UTS2 | 1 | 7927456 | 0.292 | 5' | 50K IBC |
| **rs579992** | UTS2 | 1 | 7927981 | 0.083 | 5' | 50K IBC |
| **rs6772358** | UTS2D | 3 | 190986740 | 0.362 | Intron | TaqMan |
| **rs17465071** | UTS2D | 3 | 190987067 | 0.264 | Intron | TaqMan |
| **rs9809263** | UTS2D | 3 | 190989466 | 0.333 | Intron | TaqMan |
| **rs2886062** | UTS2D | 3 | 190990163 | 0.153 | Intron | TaqMan |
| **rs4399929** | UTS2D | 3 | 190994143 | 0.306 | Intron | TaqMan |
| **rs6444532** | UTS2D | 3 | 190999760 | 0.324 | Intron | TaqMan |
| **rs6788319** | UTS2D | 3 | 19099997 | 0.333 | Exon | 50K IBC |
| **rs7502620** | UTS2R | 17 | 80320561 | 0.347 | 5' | TaqMan |
| **rs7211435** | UTS2R | 17 | 80323686 | 0.342 | 5' | TaqMan |
| **rs11650469** | UTS2R | 17 | 80330676 | 0.264 | 5' | TaqMan |
| **rs11077991** | UTS2R | 17 | 80330774 | 0.194 | 5' | TaqMan |
| **rs6502104** | UTS2R | 17 | 80331778 | 0.342 | 5' | TaqMan |

**Table S2.**

| **SNP** | **Gene** | **Chr** | **Position** | **MAF** | **Missingness (%)** | **HWE P-value** |
| --- | --- | --- | --- | --- | --- | --- |
| **rs707472** | UTS2 | 1 | 7906008 | 0.179 | 0.8 | 0.7493 |
| **rs228652** | UTS2 | 1 | 7908888 | 0.252 | 0.7 | 0.9340 |
| **rs2890565** | UTS2 | 1 | 7909737 | 0.032 | 0.7 | 0.6205 |
| **rs170629** | UTS2 | 1 | 7910391 | 0.369 | 1.4 | 0.1385 |
| **rs228651** | UTS2 | 1 | 7911099 | 0.374 | 1.3 | 0.1604 |
| **rs34305100** | UTS2 | 1 | 7913029 | 0.197 | 3.3 | 0.2372 |
| **rs228648** | UTS2 | 1 | 7913430 | 0.433 | 2.1 | 0.4409 |
| **rs13306061** | UTS2 | 1 | 7913445 | 0.200 | 0.7 | 0.0241 |
| **rs4908486** | UTS2 | 1 | 7914835 | 0.315 | 2.3 | 0.1255 |
| **rs707476** | UTS2 | 1 | 7918106 | 0.311 | 0.7 | 0.1257 |
| **rs228638** | UTS2 | 1 | 7918598 | 0.459 | 0.7 | 0.1862 |
| **rs2859389** | UTS2 | 1 | 7920423 | 0.296 | 0.7 | 0.0611 |
| **rs531485** | UTS2 | 1 | 7921952 | 0.220 | 0.7 | 0.2741 |
| **rs12566535** | UTS2 | 1 | 7924094 | 0.116 | 0.7 | 0.2216 |
| **rs500508** | UTS2 | 1 | 7927456 | 0.312 | 0.8 | 0.0412 |
| **rs579992** | UTS2 | 1 | 7927981 | 0.108 | 0.7 | 0.1416 |
| **rs6772358** | UTS2D | 3 | 190986740 | 0.384 | 3.3 | 0.1597 |
| **rs17465071** | UTS2D | 3 | 190987067 | 0.257 | 2.1 | 0.3229 |
| **rs9809263** | UTS2D | 3 | 190989466 | 0.431 | 2.7 | 0.0820 |
| **rs2886062** | UTS2D | 3 | 190990163 | 0.324 | 2.5 | 0.1145 |
| **rs4399929** | UTS2D | 3 | 190994143 | 0.267 | 2.6 | 0.6876 |
| **rs6444532** | UTS2D | 3 | 190999760 | 0.353 | 3.3 | 0.0068 |
| **rs6788319** | UTS2D | 3 | 190999917 | 0.394 | 0.7 | 0.0307 |
| **rs7502620** | UTS2R | 17 | 80320561 | 0.293 | 2.7 | 0.7036 |
| **rs7211435** | UTS2R | 17 | 80323686 | 0.296 | 2.4 | 1.0000 |
| **rs11650469** | UTS2R | 17 | 80330676 | 0.322 | 2.1 | 0.4707 |
| **rs11077991** | UTS2R | 17 | 80330774 | 0.272 | 2.8 | 0.8114 |
| **rs6502104** | UTS2R | 17 | 80331778 | 0.325 | 1.7 | 0.4311 |

**Table S3.**

| **SNP** | **Gene** | **Alleles** | **MA** | **MAF** | **Informative families** | **Z** | **P-value** |
| --- | --- | --- | --- | --- | --- | --- | --- |
| **rs707472** | UTS2 | C/A | A | 0.179 | 250 | -0.39 | 0.7002 |
| **rs228652** | UTS2 | C/T | T | 0.252 | 308 | 3.04 | 0.0024 |
| **rs2890565** | UTS2 | A/G | A | 0.032 | 63 | 1.00 | 0.3190 |
| **rs170629** | UTS2 | C/T | T | 0.369 | 368 | -2.11 | 0.0353 |
| **rs228651** | UTS2 | A/G | A | 0.374 | 367 | -2.24 | 0.0251 |
| **rs3435100** | UTS2 | C/T | C | 0.197 | 281 | -0.58 | 0.5650 |
| **rs228648** | UTS2 | C/T | C | 0.433 | 376 | 2.12 | 0.0345 |
| **rs13306061** | UTS2 | A/G | A | 0.200 | 291 | -0.25 | 0.8005 |
| **rs4908486** | UTS2 | G/A | A | 0.315 | 347 | 1.73 | 0.0828 |
| **rs707476** | UTS2 | G/A | A | 0.311 | 345 | 2.62 | 0.0087 |
| **rs228638** | UTS2 | G/T | T | 0.459 | 389 | 2.94 | 0.0033 |
| **rs2859389** | UTS2 | A/G | A | 0.296 | 341 | -3.24 | 0.0012 |
| **rs531485** | UTS2 | A/C | C | 0.220 | 291 | 3.48 | 0.0005 |
| **rs12566535** | UTS2 | T/C | T | 0.116 | 184 | 0.17 | 0.8667 |
| **rs500508** | UTS2 | A/C | A | 0.312 | 356 | -0.85 | 0.3972 |
| **rs579992** | UTS2 | A/G | G | 0.108 | 181 | -0.28 | 0.7787 |
| **rs6772358** | UTS2D | A/T | T | 0.384 | 348 | -0.04 | 0.9648 |
| **rs17465071** | UTS2D | A/G | G | 0.257 | 300 | 1.23 | 0.2179 |
| **rs9809263** | UTS2D | C/T | T | 0.431 | 353 | 0.06 | 0.9553 |
| **rs2886062** | UTS2D | G/C | G | 0.324 | 328 | 0.76 | 0.4482 |
| **rs4399929** | UTS2D | A/G | G | 0.267 | 311 | -0.01 | 0.9905 |
| **rs6444532** | UTS2D | G/T | T | 0.353 | 310 | 2.16 | 0.0308 |
| **rs6788319** | UTS2D | T/G | G | 0.394 | 344 | 2.46 | 0.0139 |
| **rs7502620** | UTS2R | A/G | A | 0.293 | 334 | -1.01 | 0.3151 |
| **rs7211435** | UTS2R | C/T | C | 0.296 | 328 | -0.62 | 0.5338 |
| **rs11650469** | UTS2R | A/G | G | 0.322 | 345 | -0.48 | 0.6337 |
| **rs11077991** | UTS2R | C/G | G | 0.272 | 321 | -0.25 | 0.8060 |
| **rs6502104** | UTS2R | A/C | C | 0.325 | 343 | -0.28 | 0.7767 |

**Table S4.**

| **SNP** | **Gene** | **Alleles** | **MA** | **MAF** | **Informative families** | **Z** | **P-value** |
| --- | --- | --- | --- | --- | --- | --- | --- |
| **rs707472** | UTS2 | C/A | A | 0.179 | 248 | 0.1 | 0.9206 |
| **rs228652** | UTS2 | C/T | T | 0.252 | 304 | 0.80 | 0.4214 |
| **rs2890565** | UTS2 | A/G | A | 0.032 | 63 | -1.36 | 0.1723 |
| **rs170629** | UTS2 | C/T | T | 0.369 | 366 | -2.71 | 0.0067 |
| **rs228651** | UTS2 | A/G | A | 0.374 | 364 | 2.59 | 0.0094 |
| **rs3435100** | UTS2 | C/T | C | 0.197 | 276 | -0.04 | 0.9645 |
| **rs228648** | UTS2 | C/T | C | 0.433 | 373 | -1.33 | 0.1832 |
| **rs13306061** | UTS2 | A/G | A | 0.200 | 287 | -0.02 | 0.9858 |
| **rs4908486** | UTS2 | G/A | A | 0.315 | 343 | -0.86 | 0.3875 |
| **rs707476** | UTS2 | G/A | A | 0.311 | 341 | -0.44 | 0.6584 |
| **rs228638** | UTS2 | G/T | T | 0.459 | 385 | 0.74 | 0.4587 |
| **rs2859389** | UTS2 | A/G | A | 0.296 | 338 | 2.05 | 0.0407 |
| **rs531485** | UTS2 | A/C | C | 0.220 | 287 | -0.16 | 0.8695 |
| **rs12566535** | UTS2 | T/C | T | 0.116 | 183 | 1.11 | 0.2652 |
| **rs500508** | UTS2 | A/C | A | 0.312 | 352 | -0.71 | 0.4757 |
| **rs579992** | UTS2 | A/G | G | 0.108 | 179 | 0.94 | 0.3449 |
| **rs6772358** | UTS2D | A/T | T | 0.384 | 344 | 0.76 | 0.4457 |
| **rs17465071** | UTS2D | A/G | G | 0.257 | 296 | -0.04 | 0.9686 |
| **rs9809263** | UTS2D | C/T | T | 0.431 | 349 | 1.14 | 0.2541 |
| **rs2886062** | UTS2D | G/C | G | 0.324 | 326 | 1.85 | 0.0648 |
| **rs4399929** | UTS2D | A/G | G | 0.267 | 308 | 1.09 | 0.2743 |
| **rs6444532** | UTS2D | G/T | T | 0.353 | 306 | -0.38 | 0.7007 |
| **rs6788319** | UTS2D | T/G | G | 0.394 | 340 | -0.11 | 0.9145 |
| **rs7502620** | UTS2R | A/G | A | 0.293 | 331 | 0.04 | 0.9661 |
| **rs7211435** | UTS2R | C/T | C | 0.296 | 325 | 0.55 | 0.5851 |
| **rs11650469** | UTS2R | A/G | G | 0.322 | 340 | -0.51 | 0.6096 |
| **rs11077991** | UTS2R | C/G | G | 0.272 | 318 | -0.39 | 0.6998 |
| **rs6502104** | UTS2R | A/C | C | 0.325 | 338 | -0.30 | 0.7616 |

**Table S5.**

| **SNP** | **Gene** | **Alleles** | **MA** | **MAF** | **Informative families** | **Z** | **P-value** |
| --- | --- | --- | --- | --- | --- | --- | --- |
| **rs707472** | UTS2 | C/A | A | 0.179 | 248 | 1.36 | 0.1735 |
| **rs228652** | UTS2 | C/T | T | 0.252 | 304 | 1.52 | 0.1276 |
| **rs2890565** | UTS2 | A/G | A | 0.032 | 63 | -1.22 | 0.2212 |
| **rs170629** | UTS2 | C/T | T | 0.369 | 366 | -2.23 | 0.0258 |
| **rs228651** | UTS2 | A/G | A | 0.374 | 364 | 1.79 | 0.0742 |
| **rs3435100** | UTS2 | C/T | C | 0.197 | 276 | 0.37 | 0.7123 |
| **rs228648** | UTS2 | C/T | C | 0.433 | 373 | -1.39 | 0.1623 |
| **rs13306061** | UTS2 | A/G | A | 0.200 | 287 | 0.25 | 0.8002 |
| **rs4908486** | UTS2 | G/A | A | 0.315 | 343 | -0.68 | 0.4997 |
| **rs707476** | UTS2 | G/A | A | 0.311 | 341 | -1.38 | 0.1682 |
| **rs228638** | UTS2 | G/T | T | 0.459 | 385 | 1.65 | 0.0989 |
| **rs2859389** | UTS2 | A/G | A | 0.296 | 338 | 2.36 | 0.0182 |
| **rs531485** | UTS2 | A/C | C | 0.220 | 287 | 1.26 | 0.2088 |
| **rs12566535** | UTS2 | T/C | T | 0.116 | 183 | 0.52 | 0.6042 |
| **rs500508** | UTS2 | A/C | A | 0.312 | 352 | 0.18 | 0.8545 |
| **rs579992** | UTS2 | A/G | G | 0.108 | 179 | -0.09 | 0.9214 |
| **rs6772358** | UTS2D | A/T | T | 0.384 | 344 | 1.15 | 0.2488 |
| **rs17465071** | UTS2D | A/G | G | 0.257 | 296 | 0.02 | 0.9828 |
| **rs9809263** | UTS2D | C/T | T | 0.431 | 349 | 2.01 | 0.0446 |
| **rs2886062** | UTS2D | G/C | G | 0.324 | 326 | 1.94 | 0.0530 |
| **rs4399929** | UTS2D | A/G | G | 0.267 | 308 | 1.25 | 0.2122 |
| **rs6444532** | UTS2D | G/T | T | 0.353 | 306 | -1.65 | 0.0988 |
| **rs6788319** | UTS2D | T/G | G | 0.394 | 340 | 1.17 | 0.2422 |
| **rs7502620** | UTS2R | A/G | A | 0.293 | 331 | -1.38 | 0.1671 |
| **rs7211435** | UTS2R | C/T | C | 0.296 | 325 | -0.90 | 0.3680 |
| **rs11650469** | UTS2R | A/G | G | 0.322 | 340 | 0.58 | 0.5617 |
| **rs11077991** | UTS2R | C/G | G | 0.272 | 318 | 0.42 | 0.6766 |
| **rs6502104** | UTS2R | A/C | C | 0.325 | 338 | 0.49 | 0.6226 |

**Table S6.**

| **SNP** | **Gene** | **Alleles** | **MA** | **MAF** | **Informative families** | **Z** | **P-value** |
| --- | --- | --- | --- | --- | --- | --- | --- |
| **rs707472** | UTS2 | C/A | A | 0.179 | 250 | -1.05 | 0.2943 |
| **rs228652** | UTS2 | C/T | T | 0.252 | 309 | -1.22 | 0.2216 |
| **rs2890565** | UTS2 | A/G | A | 0.032 | 63 | 0.76 | 0.4464 |
| **rs170629** | UTS2 | C/T | T | 0.369 | 369 | 0.64 | 0.5213 |
| **rs228651** | UTS2 | A/G | A | 0.374 | 368 | 0.31 | 0.7564 |
| **rs3435100** | UTS2 | C/T | C | 0.197 | 282 | 1.44 | 0.1493 |
| **rs228648** | UTS2 | C/T | C | 0.433 | 377 | -0.45 | 0.6565 |
| **rs13306061** | UTS2 | A/G | A | 0.200 | 292 | 1.28 | 0.1999 |
| **rs4908486** | UTS2 | G/A | A | 0.315 | 348 | 0.40 | 0.6911 |
| **rs707476** | UTS2 | G/A | A | 0.311 | 346 | -2.83 | 0.0046 |
| **rs228638** | UTS2 | G/T | T | 0.459 | 390 | -0.10 | 0.9216 |
| **rs2859389** | UTS2 | A/G | A | 0.296 | 342 | 0.92 | 0.3596 |
| **rs531485** | UTS2 | A/C | C | 0.220 | 291 | -1.84 | 0.0660 |
| **rs12566535** | UTS2 | T/C | T | 0.116 | 184 | 0.36 | 0.7206 |
| **rs500508** | UTS2 | A/C | A | 0.312 | 357 | 0.51 | 0.6084 |
| **rs579992** | UTS2 | A/G | G | 0.108 | 182 | -1.33 | 0.1842 |
| **rs6772358** | UTS2D | A/T | T | 0.384 | 348 | 0.11 | 0.9093 |
| **rs17465071** | UTS2D | A/G | G | 0.257 | 301 | -0.95 | 0.3441 |
| **rs9809263** | UTS2D | C/T | T | 0.431 | 353 | 1.12 | 0.2611 |
| **rs2886062** | UTS2D | G/C | G | 0.324 | 328 | 0.19 | 0.8477 |
| **rs4399929** | UTS2D | A/G | G | 0.267 | 312 | 0.33 | 0.7437 |
| **rs6444532** | UTS2D | G/T | T | 0.353 | 310 | -0.06 | 0.9533 |
| **rs6788319** | UTS2D | T/G | G | 0.394 | 345 | 0.06 | 0.9505 |
| **rs7502620** | UTS2R | A/G | A | 0.293 | 328 | 0.24 | 0.8095 |
| **rs7211435** | UTS2R | C/T | C | 0.296 | 322 | 0.69 | 0.4909 |
| **rs11650469** | UTS2R | A/G | G | 0.322 | 338 | 0.67 | 0.5008 |
| **rs11077991** | UTS2R | C/G | G | 0.272 | 314 | 0.88 | 0.3810 |
| **rs6502104** | UTS2R | A/C | C | 0.325 | 336 | 1.04 | 0.2986 |

**Table S7.**

| **SNP** | **Gene** | **Alleles** | **MA** | **MAF** | **Informative families** | **Z** | **P-value** |
| --- | --- | --- | --- | --- | --- | --- | --- |
| **rs707472** | UTS2 | C/A | A | 0.179 | 250 | -0.21 | 0.8369 |
| **rs228652** | UTS2 | C/T | T | 0.252 | 309 | 0.57 | 0.5683 |
| **rs2890565** | UTS2 | A/G | A | 0.032 | 63 | 1.72 | 0.0861 |
| **rs170629** | UTS2 | C/T | T | 0.369 | 369 | -0.70 | 0.4854 |
| **rs228651** | UTS2 | A/G | A | 0.374 | 368 | -0.64 | 0.5199 |
| **rs3435100** | UTS2 | C/T | C | 0.197 | 282 | -0.43 | 0.6672 |
| **rs228648** | UTS2 | C/T | C | 0.433 | 377 | -0.57 | 0.5686 |
| **rs13306061** | UTS2 | A/G | A | 0.200 | 292 | -0.32 | 0.7527 |
| **rs4908486** | UTS2 | G/A | A | 0.315 | 348 | -0.40 | 0.6915 |
| **rs707476** | UTS2 | G/A | A | 0.311 | 346 | -0.42 | 0.6730 |
| **rs228638** | UTS2 | G/T | T | 0.459 | 390 | 0.34 | 0.7345 |
| **rs2859389** | UTS2 | A/G | A | 0.296 | 342 | -0.78 | 0.4332 |
| **rs531485** | UTS2 | A/C | C | 0.220 | 291 | 0.46 | 0.6464 |
| **rs12566535** | UTS2 | T/C | T | 0.116 | 184 | 1.16 | 0.2468 |
| **rs500508** | UTS2 | A/C | A | 0.312 | 357 | -0.93 | 0.3512 |
| **rs579992** | UTS2 | A/G | G | 0.108 | 182 | -0.68 | 0.4939 |
| **rs6772358** | UTS2D | A/T | T | 0.384 | 348 | 1.25 | 0.2125 |
| **rs17465071** | UTS2D | A/G | G | 0.257 | 301 | -1.80 | 0.0713 |
| **rs9809263** | UTS2D | C/T | T | 0.431 | 353 | 2.39 | 0.0170 |
| **rs2886062** | UTS2D | G/C | G | 0.324 | 328 | 0.81 | 0.4164 |
| **rs4399929** | UTS2D | A/G | G | 0.267 | 312 | 0.86 | 0.3875 |
| **rs6444532** | UTS2D | G/T | T | 0.353 | 310 | -1.42 | 0.1567 |
| **rs6788319** | UTS2D | T/G | G | 0.394 | 345 | -1.36 | 0.1729 |
| **rs7502620** | UTS2R | A/G | A | 0.293 | 334 | 0.28 | 0.7807 |
| **rs7211435** | UTS2R | C/T | C | 0.296 | 328 | 0.25 | 0.8043 |
| **rs11650469** | UTS2R | A/G | G | 0.322 | 346 | 0.15 | 0.8830 |
| **rs11077991** | UTS2R | C/G | G | 0.272 | 322 | 0.20 | 0.8393 |
| **rs6502104** | UTS2R | A/C | C | 0.325 | 344 | 0.19 | 0.8481 |

**Table S8.**

| **SNP** | **Gene** | **Alleles** | **MA** | **MAF in parents** | **Informative families** | **Z** | **P-value** |
| --- | --- | --- | --- | --- | --- | --- | --- |
| **rs707472** | UTS2 | C/A | A | 0.179 | 250 | -1.27 | 0.2026 |
| **rs228652** | UTS2 | C/T | T | 0.252 | 309 | -0.62 | 0.5324 |
| **rs2890565** | UTS2 | A/G | A | 0.032 | 63 | -0.87 | 0.3842 |
| **rs170629** | UTS2 | C/T | T | 0.369 | 369 | 1.07 | 0.2857 |
| **rs228651** | UTS2 | A/G | A | 0.374 | 368 | 1.09 | 0.2746 |
| **rs3435100** | UTS2 | C/T | C | 0.197 | 282 | 0.34 | 0.7343 |
| **rs228648** | UTS2 | C/T | C | 0.433 | 377 | -0.94 | 0.3471 |
| **rs13306061** | UTS2 | A/G | A | 0.200 | 292 | 0.28 | 0.7775 |
| **rs4908486** | UTS2 | G/A | A | 0.315 | 348 | -0.13 | 0.8998 |
| **rs707476** | UTS2 | G/A | A | 0.311 | 346 | -2.17 | 0.0303 |
| **rs228638** | UTS2 | G/T | T | 0.459 | 390 | -0.50 | 0.6208 |
| **rs2859389** | UTS2 | A/G | A | 0.296 | 342 | 1.28 | 0.2018 |
| **rs531485** | UTS2 | A/C | C | 0.220 | 291 | -0.81 | 0.4158 |
| **rs12566535** | UTS2 | T/C | T | 0.116 | 184 | 0.50 | 0.6208 |
| **rs500508** | UTS2 | A/C | A | 0.312 | 357 | -1.01 | 0.3129 |
| **rs579992** | UTS2 | A/G | G | 0.108 | 182 | -2.05 | 0.0402 |
| **rs6772358** | UTS2D | A/T | T | 0.384 | 348 | -1.00 | 0.3173 |
| **rs17465071** | UTS2D | A/G | G | 0.257 | 353 | -0.88 | 0.3776 |
| **rs9809263** | UTS2D | C/T | T | 0.431 | 328 | -1.24 | 0.2145 |
| **rs2886062** | UTS2D | G/C | G | 0.324 | 312 | 1.34 | 0.1817 |
| **rs4399929** | UTS2D | A/G | G | 0.267 | 310 | -1.47 | 0.1406 |
| **rs6444532** | UTS2D | G/T | T | 0.353 | 301 | -0.40 | 0.6866 |
| **rs6788319** | UTS2D | T/G | G | 0.394 | 345 | -1.64 | 0.1006 |
| **rs7502620** | UTS2R | A/G | A | 0.293 | 334 | -1.36 | 0.1739 |
| **rs7211435** | UTS2R | C/T | C | 0.296 | 328 | -0.92 | 0.3591 |
| **rs11650469** | UTS2R | A/G | G | 0.322 | 346 | 1.17 | 0.2424 |
| **rs11077991** | UTS2R | C/G | G | 0.272 | 322 | 0.10 | 0.9247 |
| **rs6502104** | UTS2R | A/C | C | 0.325 | 344 | 1.13 | 0.2598 |

**Table S9.**

| **SNP** | **Gene** | **Alleles** | **MA** | **MAF** | **Informative families** | **Z** | **P-value** |
| --- | --- | --- | --- | --- | --- | --- | --- |
| **rs707472** | UTS2 | C/A | A | 0.179 | 250 | -0.31 | 0.7575 |
| **rs228652** | UTS2 | C/T | T | 0.252 | 309 | 0.42 | 0.6714 |
| **rs2890565** | UTS2 | A/G | A | 0.032 | 63 | 0.77 | 0.4419 |
| **rs170629** | UTS2 | C/T | T | 0.369 | 369 | -1.01 | 0.3131 |
| **rs228651** | UTS2 | A/G | A | 0.374 | 368 | -0.83 | 0.4055 |
| **rs3435100** | UTS2 | C/T | C | 0.197 | 282 | 0.76 | 0.4463 |
| **rs228648** | UTS2 | C/T | C | 0.433 | 377 | 0.32 | 0.7509 |
| **rs13306061** | UTS2 | A/G | A | 0.200 | 292 | 0.59 | 0.5585 |
| **rs4908486** | UTS2 | G/A | A | 0.315 | 348 | 0.97 | 0.3301 |
| **rs707476** | UTS2 | G/A | A | 0.311 | 346 | 0.45 | 0.6563 |
| **rs228638** | UTS2 | G/T | T | 0.459 | 390 | 1.91 | 0.0566 |
| **rs2859389** | UTS2 | A/G | A | 0.296 | 342 | -1.95 | 0.0515 |
| **rs531485** | UTS2 | A/C | C | 0.220 | 291 | 2.05 | 0.0405 |
| **rs12566535** | UTS2 | T/C | T | 0.116 | 184 | 1.10 | 0.2718 |
| **rs500508** | UTS2 | A/C | A | 0.312 | 357 | -0.30 | 0.7674 |
| **rs579992** | UTS2 | A/G | G | 0.108 | 182 | -1.26 | 0.2091 |
| **rs6772358** | UTS2D | A/T | T | 0.384 | 348 | -1.04 | 0.2982 |
| **rs17465071** | UTS2D | A/G | G | 0.257 | 301 | -0.22 | 0.8245 |
| **rs9809263** | UTS2D | C/T | T | 0.431 | 353 | -0.24 | 0.8109 |
| **rs2886062** | UTS2D | G/C | G | 0.324 | 328 | -0.99 | 0.3227 |
| **rs4399929** | UTS2D | A/G | G | 0.267 | 312 | 1.69 | 0.0908 |
| **rs6444532** | UTS2D | G/T | T | 0.353 | 310 | -2.00 | 0.0461 |
| **rs6788319** | UTS2D | T/G | G | 0.394 | 345 | -1.68 | 0.0933 |
| **rs7502620** | UTS2R | A/G | A | 0.293 | 334 | -0.27 | 0.7906 |
| **rs7211435** | UTS2R | C/T | C | 0.296 | 328 | -0.23 | 0.8198 |
| **rs11650469** | UTS2R | A/G | G | 0.322 | 346 | 1.41 | 0.1599 |
| **rs11077991** | UTS2R | C/G | G | 0.272 | 322 | 0.51 | 0.6139 |
| **rs6502104** | UTS2R | A/C | C | 0.325 | 344 | 1.32 | 0.1868 |

**Table S10.**

| **SNP** | **Gene** | **MA** | **MAF** | **β** | **SE** | **P-value** |
| --- | --- | --- | --- | --- | --- | --- |
| **rs228652** | UTS2 | T | 0.283 | -0.92 | 0.59 | 0.1231 |
| **rs170629** | UTS2 | T | 0.368 | 0.10 | 0.59 | 0.8592 |
| **rs228651** | UTS2 | A | 0.385 | 0.35 | 0.58 | 0.5447 |
| **rs3435100** | UTS2 | C | 0.183 | -0.27 | 0.71 | 0.6994 |
| **rs228648** | UTS2 | C | 0.418 | -0.48 | 0.53 | 0.3655 |
| **rs13306061** | UTS2 | A | 0.172 | -0.21 | 0.71 | 0.7656 |
| **rs2859389** | UTS2 | A | 0.305 | 0.71 | 0.59 | 0.2343 |
| **rs531485** | UTS2 | C | 0.283 | -0.46 | 0.54 | 0.3899 |
| **rs6772358** | UTS2D | T | 0.344 | -0.14 | 0.22 | 0.5312 |
| **rs17465071** | UTS2D | G | 0.361 | 0.23 | 0.22 | 0.2943 |
| **rs9809263** | UTS2D | T | 0.385 | -0.05 | 0.22 | 0.7965 |
| **rs2886062** | UTS2D | G | 0.295 | -0.05 | 0.25 | 0.8282 |
| **rs4399929** | UTS2D | G | 0.238 | 0.64 | 0.26 | 0.0176 |
| **rs6444532** | UTS2D | T | 0.336 | -0.18 | 0.21 | 0.3658 |
| **rs7502620** | UTS2R | A | 0.245 | 0.82 | 0.74 | 0.2761 |
| **rs7211435** | UTS2R | C | 0.250 | 0.78 | 0.75 | 0.3016 |
| **rs11650469** | UTS2R | G | 0.328 | 0.39 | 0.64 | 0.5507 |
| **rs11077991** | UTS2R | G | 0.287 | 0.03 | 0.64 | 0.9629 |
| **rs6502104** | UTS2R | C | 0.328 | 0.39 | 0.64 | 0.5504 |

**Figure S1.**


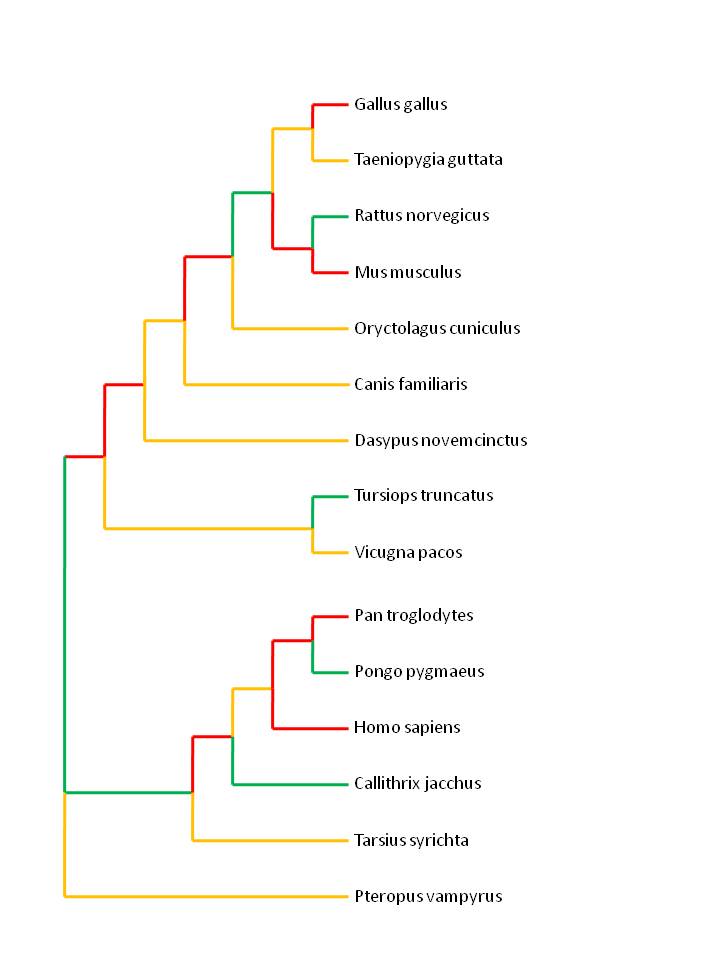


**Figure S2.**


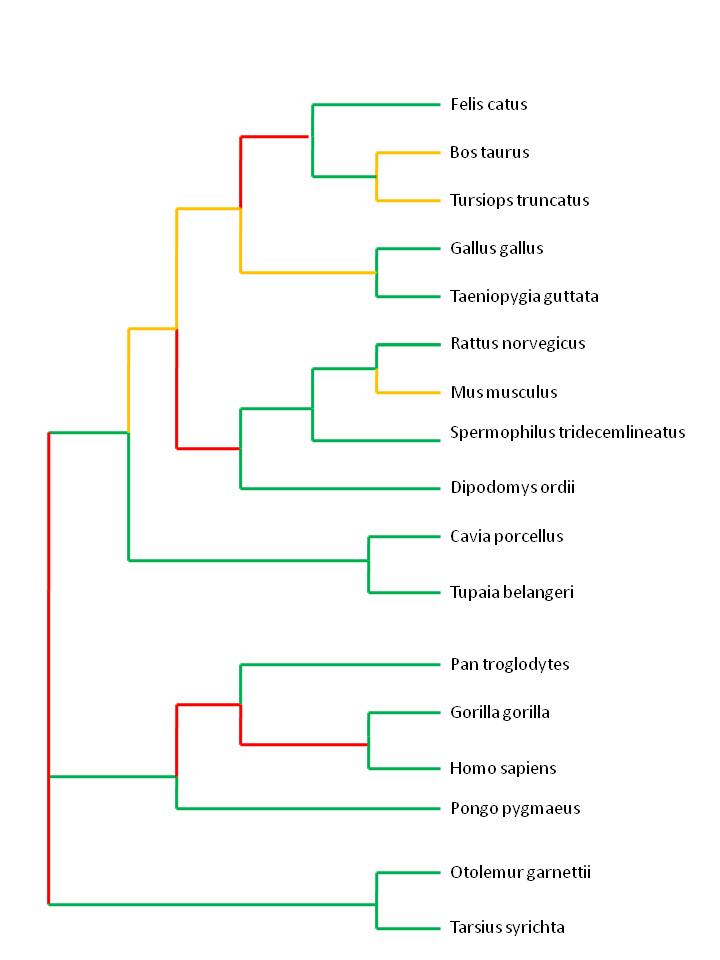


**Figure S3.**


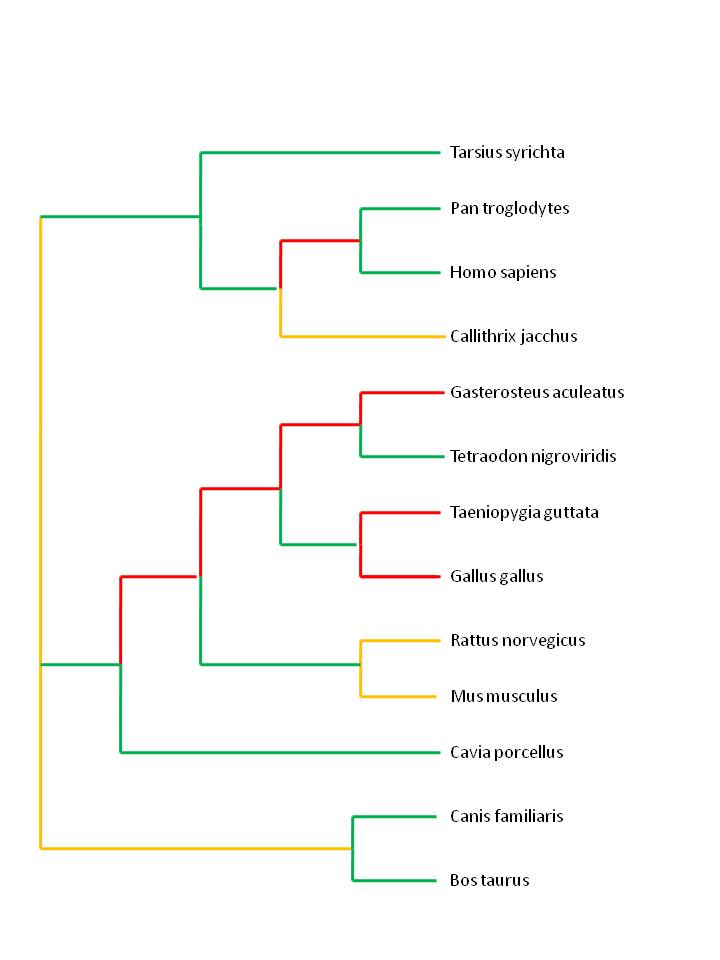


**References**

1. Keating BJ, Tischfield S, Murray SS, Bhangale T, Price TS, et al. (2008) Concept, Design and Implementation of a Cardiovascular Gene-Centric 50 K SNP Array for Large-Scale Genomic Association Studies. PLoS One 3: e3583.

2. Nyholt DR (2004) A simple correction for multiple testing for single-nucleotide polymorphisms in linkage disequilibrium with each other. Am J Hum Genet. 74: 765-769.

3. Manolio TA, Brooks LD, Collins FS (2008) A HapMap harvest of insights into the genetics of common disease. J Clin Invest. 118: 1590-1605.

4. Bender R, Lange S (1999) Multiple test procedures other than Bonferroni's deserve wider use. BMJ 318: 600-601.
